# The Stem Cell-Expressed Receptor Lgr5 Possesses Canonical and Functionally Active Molecular Determinants Critical to β-arrestin-2 Recruitment

**DOI:** 10.1371/journal.pone.0084476

**Published:** 2013-12-27

**Authors:** Joshua C. Snyder, Lauren K. Rochelle, Larry S. Barak, Marc G. Caron

**Affiliations:** 1 Department of Cell Biology, Duke University Medical Center, Durham, North Carolina, United States of America; 2 Department of Neurobiology, Duke University Medical Center, Durham, North Carolina, United States of America; 3 Department of Medicine, Duke University Medical Center, Durham, North Carolina, United States of America; University of North Dakota, United States of America

## Abstract

Lgr5 is a membrane protein related to G protein-coupled receptors (GPCR)s whose expression identifies stem cells in multiple tissues and is strongly correlated with cancer. Despite the recent identification of endogenous ligands for Lgr5, its mode of signaling remains enigmatic. The ability to couple to G proteins and βarrestins are classical molecular behaviors of GPCRs that have yet to be observed for Lgr5. Therefore, the goal of this study was to determine if Lgr5 can engage a classical GPCR behavior and elucidate the molecular determinants of this process. Structural analysis of Lgr5 revealed several motifs consistent with its ability to recruit βarr2. Among them, a “SSS” serine cluster located at amino acid position 873-875 within the C-terminal tail (C-tail), is in a region consistent with other GPCRs that bind βarr2 with high-affinity. To test its functionality, a ligand-independent βarr2 translocation assay was implemented. We show that Lgr5 recruits βarr2 and that the “SSS” amino acids (873-875) are absolutely critical to this process. We also demonstrate that for full efficacy, this cluster requires other Lgr5 C-tail serines that were previously shown to be important for constitutive and βarr2 independent internalization of Lgr5. These data are proof of principle that a classical GPCR behavior can be manifested by Lgr5. The existence of alternative ligands or missing effectors of Lgr5 that scaffold this classical GPCR behavior and the downstream signaling pathways engaged should be considered. Characterizing Lgr5 signaling will be invaluable for assessing its role in tissue maintenance, repair, and disease.

## Introduction

Lgr5 belongs to a family of G protein-coupled receptors (GPCRs^*^) termed leucine-rich GPCRs (Lgr)s [1,2]. A subfamily of these receptors, include Lgr4-6, which possess large N-terminal extracellular domains that contain varying numbers of leucine-rich repeats (LRR)s. Lgr4-6 are classified as rhodopsin-like Class 1 GPCRs, because, despite their relatively oversized N-termini, they also possess a seven transmembrane (7-TM) domain structure typical of GPCRs in general [3]. In the case of Lgr1-3 (FSHR, LHR, and TSHR, respectively), these N-terminal domains bind to heterodimeric cystine-knot protein hormones, and the receptors themselves couple to G_αs_ to stimulate cAMP production [4] and also engage βarr2 [5-7]. 

 In contrast to Lgr1-3, the biochemical and cellular details of signal transduction for Lgr4-6 are only beginning to emerge. Since the discovery of Lgr4-6, their functions have remained elusive, due in part to an orphan status, meaning that functionally active cognate ligands remain unknown. In 2007, Barker et al demonstrated, by lineage tracing, that Lgr5 expression can be used to identify epithelial stem cells of the small and large intestine [8]. Since this discovery, Lgr5 mediated lineage tracing has reliably identified stem cells in several other tissues and interestingly Lgr5 expression has been correlated with cancer [9-11]. These findings fueled the search for cognate Lgr4-6 ligands and their signaling mechanism. In 2011 and 2012 several groups reported high affinity interactions of Lgr4-6 with Rspondins1-4 [12-16]. More recently, the cystine knot protein, Norrin, was also shown to interact with Lgr4 [17]. 

Incredibly, none of these high-affinity ligands seem able to induce classical GPCR behaviors such as coupling to one of the diverse G-proteins or engagement of the βarrs. Ligand mediated activation of receptors and their subsequent phosphorylation by G protein-coupled receptor kinases (GRK)s stimulate the recruitment of βarrs [18]. Once recruited, βarrs regulate GPCR desensitization and endocytosis but also function as G protein independent signaling scaffolds [19-23]. The inability to demonstrate that the high affinity Lgr4-6 ligands can direct either of these two GPCR behaviors has led some to even question whether Lgr4-6 are indeed GPCRs and therefore should be reclassified [10]. Thus, answering this fundamental question is of great importance in order to gain insight into the Lgr4-6 driven signaling program in stem cell and cancer cell behavior, as well as implementing drug discovery programs targeting this unique class of receptors.

To answer this question, we were aided by previous work in which the molecular determinants that mediate the constitutive internalization of Lgr5 to the trans-Golgi network were discovered. This unique property of Lgr5 was utilized in a series of structure and function studies, which revealed a serine motif in the C-tail situated between amino acids 844 and 864 modulates this process. In particular, serine residues at position 861 and 864 were found to be critical to this phenomenon. A serine cluster “SSS” (aa873-875) downstream from this motif appears similar to those present in many other GPCRs that bind βarr2 and is a predicted GRK substrate, that surprisingly, wasn’t necessary for Lgr5 internalization [24]. These data suggested that constitutive internalization occurs independent of βarr2. However, whether this “SSS” cluster is a conserved βarr2 recruitment motif that possesses any functional activity wasn’t tested.

On this basis, we sought to determine if Lgr4-6, and in particular Lgr5, harbor protein motifs consistent with their ability to display classical GPCR behaviors. Lgr4-6 primary amino acid sequences were interrogated for the existence of molecular determinants conserved in other Class 1 GPCRs, which mediate classical GPCR behavior. We found that the aforementioned “SSS” cluster present in Lgr5 is a hypothetical high-affinity βarr2 motif. Importantly, we demonstrate that Lgr5 can recruit βarr2 in a GRK-dependent manner and that the “SSS” motif is fully functional and critical to this process. These data provide proof of principle that Lgr5 can engage classical GPCR behaviors. These results provide an important first step in assessing Lgr5 as a functional GPCR. Therefore, continued studies aimed at the discovery of Lgr5 orthosteric ligands and effectors supportive of classical GPCR behavior are warranted.

## Materials and Methods

### Cloning and Plasmids

Constructs encoding the Renilla reinformis green fluorescent protein (GFP) fused to the C-terminus of *rattus norvegicus* β-arrestin-2 (βarr2-GFP) or GFP fused to the C-terminus of βarr1 (βarr1-GFP) were previously described [25,26]. GRK2 (*Bos taurus NM_174710*), GRK4 (*Homo sapiens NM_182982*) , GRK5 (*Bos taurus NM_174331*), and GRK6 (*Homo sapiens* NM_001004015) were available in the lab. The 3xHA-N-terminal epitope tagged human Lgr5 with an EGFP fused in-frame at the C-terminus and phosphor-acceptor mutants were described previously [24]. Using these constructs, a stop sequence was inserted between the C-tail and EGFP fusion to eliminate EGFP expression and make these constructs amenable to the βarr2-GFP translocation assay. Additional mutants not described in previous work were also generated using PCR mutagenesis, as previously described [24].

### Clustal Alignment

**Figure 1:**
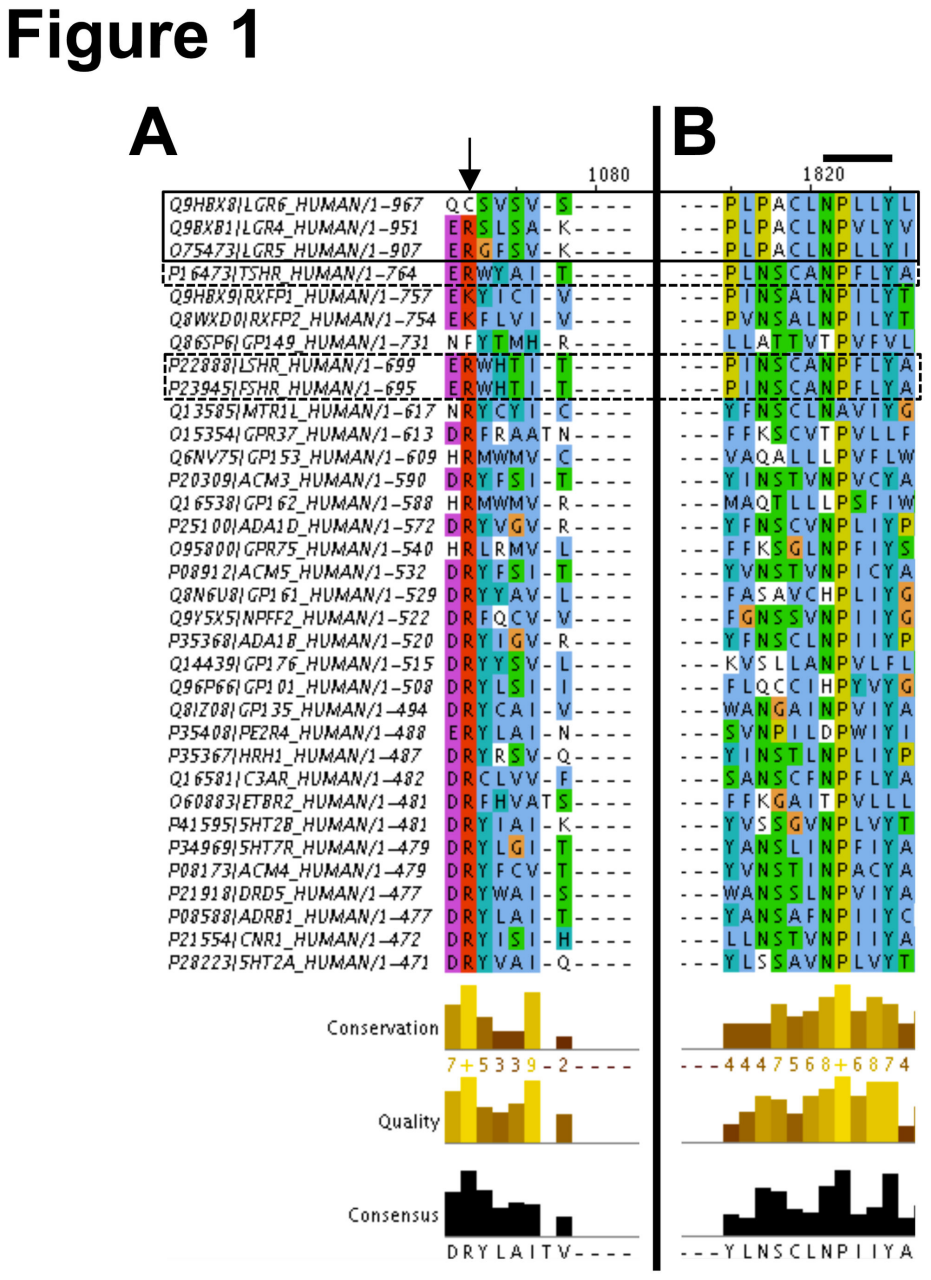
Conservation of typical GPCR signaling motifs in Lgr4-6. The primary amino acid sequences of 282 Class 1 rhodopsin-like GPCRs were aligned in ClustalΩ and a subset presented due to space limitations (ClustalX color view based upon degree of conservation; See jalview.org for complete color table, briefly, Acidic (E,D: Magenta); Basic (R, K:Red); (P: Yellow). The entire sequence alignment can be downloaded from File S1 and opened in Jalview. Lgr1-3 (FSHR, LSHR, and TSHR) and Lgr4-6 are highlighted by hatched and solid boxes. (A) Primary acid alignment from a subset of GPCRs at the “DRY” motif and (B) primary acid alignment from a subset of GPCRs at the NPXXY motif. Arrow in (A) points to the conserved Arginine in the “DRY” motif. Line in (B) highlights the NPXXY motif. The position label at top indicates amino acid position from the start of the alignment. At the bottom, alignment conservation, quality, and consensus for each amino acid (+, indicates significant conservation of the site).

The primary amino acid sequences of Human class 1 rhodopsin-like GPCRs were retrieved from the International Union of Basic and Clinical Pharmacology (IUPHAR) database [27] (http://www.iuphardb.org/DATABASE/GPCRListForward). Sequence alignments were generated using the ClustalΩ online web applet [28,29] (http://www.ebi.ac.uk/services/proteins). Data were downloaded and uploaded into the freely available Jalview14.0 (http://www.jalview.org, downloaded and installed on Mac OSX, Cupertino, CA) for post analysis sequence visualization, sorting, and presentation [30]. Color coding of amino acid sequences was applied through ClustalX coloration and a consensus logo applied to calculate amino acid frequency for each conserved residue. Due to publishing constrains, only a subset of the receptors and their sequences in outlined in Figure 1. The complete data set is available as File S1 to be opened and viewed in Jalview. For analysis of C-tails only, full length sequences were downloaded from http://www.uniprot.org in FASTA format, C-tail sequence isolated using the NPXXY motif as an identifier, uploaded and processed by ClustalΩ, and visualized in Jalview 14.0, as described above.

### βarr2 Confocal Translocation Assays

150,000 HEK 293 T/17 cells (HEK) (ATCC CRL-11268) cultured in DMEM (Mediatech/Cellgro 10-013-CV), 10%FBS (Sigma F2242), and Antibiotic-Antimycotic (Invitrogen 15240-062) were plated in each well of 24-well glass bottom plates (MatTek Coporation, Ashton, MA (part# P24G-0-10-F)) that were previously coated with with 75μg/ml fibronectin in H_2_O (Sigma F2006). The next day cells were transfected with βarr2-GFP (0.75μg) plus the indicated receptor (1μg) and GRK (1μg) using a calcium phosphate transfection procedure [31] that was scaled back to 45μl H_2_O, 5μl 2.5M CaCl_2,_ 50μl 2xHBS per well. The next day cell media was changed to live cell staining media (SM) comprised of Clear MEM (Invitrogen 51200) plus 10mM HEPES (Invitrogen 15630) and 1xGlutaMAX-1 (Invitrogen 35050) or fixed with 4% paraformaldehyde for confocal imaging. Phosphor-acceptor mutants were blindly assessed for their ability to recruit βarr2 over a minimum of three fields.

### Antibody Staining and Pulse-Chase Internalization

Pulse-chase internalization assays were performed as previously reported [24]. Briefly, 350,000 HEK cells were plated on 12 well MatTek plates (MatTek Corporation, Ashton, MA P12G-0-10-F) that were coated with 75μg/ml fibronectin in H_2_O (Sigma F2006). Cells were transfected the next day with indicated constructs using 0.75μg βarr2-GFP, 1μg receptor, and 1μg GRK using the same protocol described above. The next day cells were washed once with ice cold SM and pulsed with a MsαHA (from a mouse hybridoma available in the lab) at 1:500 in SM for 45 minutes, washed four times with SM, and fixed with 4% paraformaldehyde or chased for 2 hours in a 37°C cell culture incubator and then fixed similarly. Cells were permeabilized for ten minutes with 0.12% Triton-x-100/PBS and then blocked with 5%BSA/0.12%Triton-x-100 for thirty minutes. Cells were then stained with GαM-568 (Invitrogen A11004) 1:1000 in blocking buffer for 1.5 hours. Cells were washed with PBS and then imaged by confocal microscopy. Antisera recognizing GRK5 and 6 (rabbit polyclonal made in house) or GRK6 specifically [Santa Cruz C20 (sc-566)] were kind gifts of Dr. Richard Premont (Figure S1).

### Confocal Analysis

A Zeiss LSM 510 confocal microscope (Carl Zeiss Microimaging) equipped with appropriate laser lines and filter sets for 568 nm, and 633 nm fluorescence imaging, was utilized to collect images. Images were acquired using a 100X objective and digital zoom set to 2x. A minimum of 3 images were collected for each sample.

### ArrestinZoom Assay

35,000 HEK cells were plated onto a 96-well tissue culture plate (Thermoscientific 167008). A transfection master-mix was prepared by mixing +/- 1μg receptor, 0.75μg βarr2-GFP, and 1μg GRK6 plasmid DNA in 90μl H_2_O, 10 μl 2.5M CaCl_2,_ and 100μl 2xHBS, for a final volume of approximately 200μl, enabled up to 8 technical replicate transfections (25μl/well). Cells were transfected one day post plating and were cultured overnight. The next day, cells were fixed with 4% paraformaldehyde for ten minutes and then washed with PBS and stored at 4°C. High-content large-field fluorescence imaging was performed using an Axiozoom.V16 (Carl Zeiss Microimaging). βarr2-GFP images were acquired with a GFP-filter set (Filter set 38 HE: *Ex* BP 470/40, *BS* FT 495, *Em* BP 525/50) at 108X magnification using the 2.3x Lens. For each well of the 96-well plate, 32 tiled-images (10% overlap between images) were acquired using Zen 2012 Blue Edition (Carl Zeiss Microimaging). Using these parameters, 25% of the well surface was imaged for each well of a 96 well plate (totaling 3072 images or 1536 images, when 96 or 48 wells were respectively imaged). Imaging acquisition time was approximately 20 minutes for an entire 96-well plate and only 10 minutes per 48-wells. A single stitched image was generated using the stitching function in Zen 2012 blue. For image processing, single high resolution non-compressed Tiff files were exported for each stitched well and imported into ImageJ [32]. Contrast and brightness were adjusted across the entire stitched image based upon the negative (βarr2-GFP and GRK6) and positive control wells (Wild-type Lgr5, βarr2-GFP, and GRK6). An image montage was created in ImageJ to reconstruct the 96-well plate format, a threshold was set to discriminate bright vesicular aggregates that formed in positive cells, and particles were analyzed and counted that had a minimum radius >1.2 pixels. Results were exported to Excel, and the number of vesicular aggregates totaled for a well in a Microsoft Excel pivot table. Bar graphs were generated in GraphPad Prism 5.0 (GraphPad Software) and statistically significant changes evaluated using a 1-way ANOVA and *post-hoc* Bonferroni analysis (p < 0.05). A minimum of 4 technical replicates for each experimental condition were included in each of the four independently performed experiments.

## Results

### Lgr4-6 possess classical GPCR structural motifs

The structural requirements for coupling to G proteins or βarrs are contained within the receptor seven transmembrane domain (7-TM), intra-cellular loops, and the C-tail. Two of the most well-studied determinants include the DRY motif located in intracellular loop 2 (IC2) and the NPXXY motif immediately proximal to the C-tail at the end of transmembrane-7 (TM7) [33-37]. To probe for the existence of these motifs in Lgr4-6, ClustalΩ [28,29] analysis of the primary amino acid sequences of 282 human class 1 rhodopsin-like GPCRs was performed. Lgr1-3 (FSHR, LHR, and TSHR) and Lgr4-6 have substantial conservation of one or both of these motifs (Figure 1, hatched and solid boxes, respectively). In particular, Lgr4 and Lgr5 have substantial conservation of the critically important arginine (conserved in 93% of the receptors clustered) in the “DRY” motif (Figure 1A *, arrow*). A proline, nine amino acids downstream of the DRY motif, aids in high affinity βarr2 binding. Consistent with previous reports, proline is conserved in 64% of rhodopsin-like GPCRs and conducive to a βarr2 interaction (File S1) [35]. Lgr4 and Lgr5 instead have an isoleucine or serine at this position. Interestingly, a proline at this position isn’t absolutely critical for βarr2 coupling, as the α_1B_-adrenergic receptor also harbors a serine and still interacts with βarr2 [38]. Lgr1-6 all have robust conservation of the “NPXXY” important for G protein coupling and engaging arrestin [34], including the consensus asparagine, proline and tyrosine, conserved in 70%, 93%, and 83% of clustered receptors (Figure 1B *, bold line*). These results suggested that Lgr4-6 harbor *bona fide* structural determinants necessary for classical GPCR behaviors warranting a thorough search for additional motifs and characterization of their functional activity.

### The βarr2 interaction motif found in the V2R is conserved in Lgr5 but not Lgr4 or Lgr6

**Figure 2:**
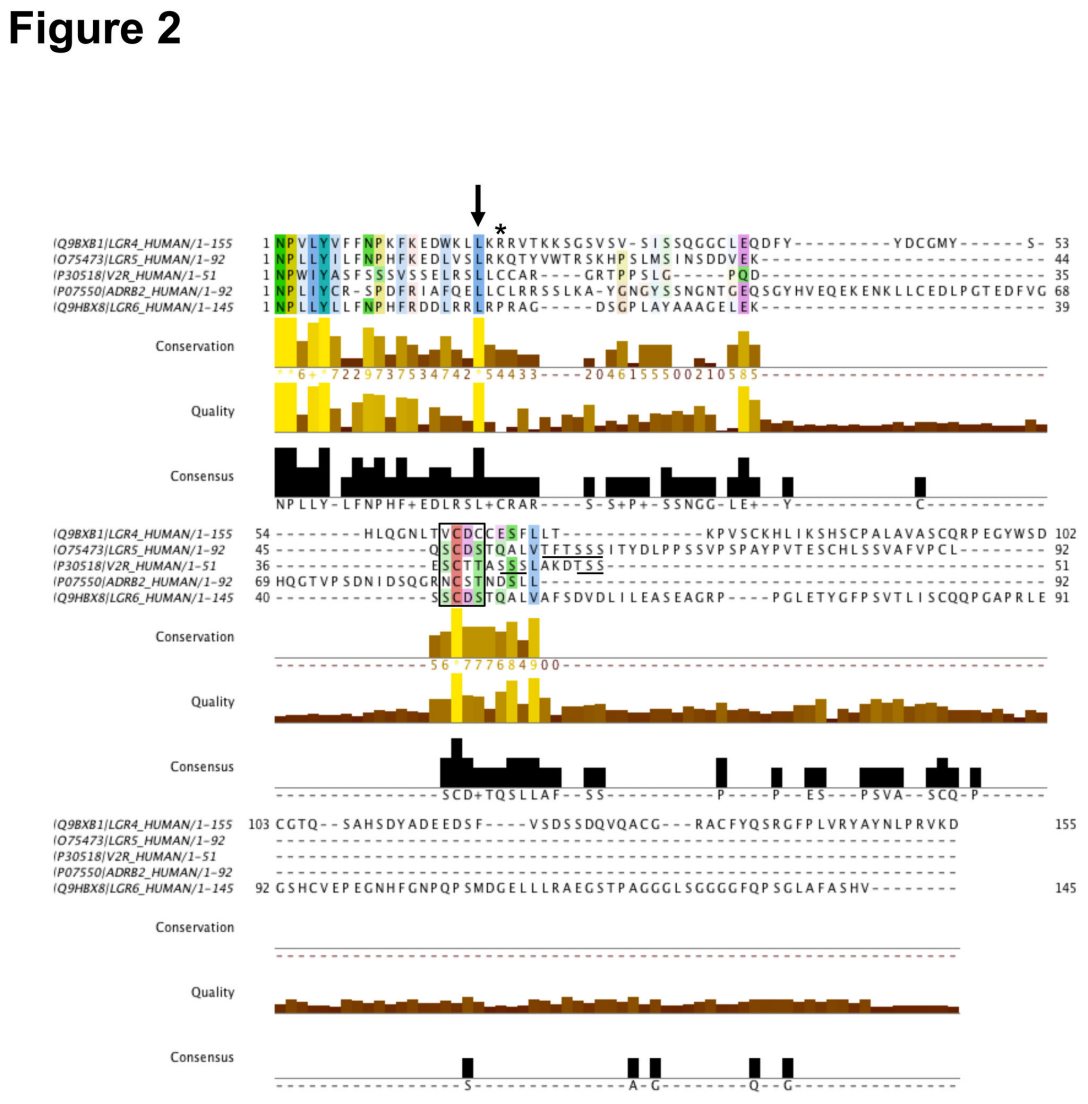
Conservation of a high-affinity interaction motif in Lgr5 but not Lgr4 or Lgr6. The C-terminal of tails of human Lgr4, Lgr5, Lgr6, V2R, and β2AR (Uniprot IDs: Q9BXB1, O75473, Q9HBX8, P30518, and P07550) were aligned in ClustalΩ starting with the conserved NPXXY motif at the end of transmembrane seven (Clustal X color view based upon degree of conservation, See jalview.org for complete color table). Asterisk and arrow, respectively denote the position of the conserved palmitoylated cysteine (absent in Lgr4/5/6) and leucine. Underlined residues identify serine/threonine clusters which are necessary for Class B βarr2 affinity of the V2R. Of particular interest, is the conservation of a similar motif in Lgr5 (aa873-5) but not Lgr4, Lgr6, or β2AR. The boxed residues represent conservation of the motif necessary for Lgr5 internalization that are also seen in the V2R and β2AR.

Upon ligand-mediated activation, GPCRs are phosphorylated by GRKs in the intracellular loops or in the C-tail to mediate the recruitment of βarr2 [18,19]. The strength and duration of this interaction varies among receptors and includes the formation of receptor/arrestin complexes transiently at the cell membrane or a long-term interaction stable into receptor/arrestin intracellular endocytic vesicles. Receptors harboring distinct modes of βarr2 recruitment have been classified as Class A and Class B, respectively [38]. The motifs necessary for Class B interactions are characterized by clusters of serine and threonine residues in the receptor tails downstream from the NPXXY motif and are substrates of GRKs [26,39,40]. Intriguingly a similar motif has already been characterized in the Lgr4-6 related human FSHR [5]. To more thoroughly determine if such functional motifs existed in Lgr4-6, ClustalΩ [28,29] analysis of the C- tails of these receptors alongside the Class A and Class B stereotypes, β2AR and V2R, was performed (Figure 2). The V2R “SSS” and “TSS” motifs (Figure 2, underlined) have been well studied, with the “SSS” absolutely critical for Class B βarr2 recruitment [40]. Interestingly, only Lgr5 possesses a threonine and serine cluster, “TFTSSS” (Figure 2, *underlined*) that also aligns with a serine cluster in V2R. Previous work from our laboratory has demonstrated that activity of the serine/threonine clusters is dependent upon their distance from an evolutionarily conserved palmitoylated cysteine (Figure 2 *) [41]. Lgr5 doesn’t possess a conserved cysteine, however, the neighboring leucine is conserved (Figure 2, *arrow*) and when used as a landmark we found the “TFTSSS” motif to be present in approximately the correct location within the C-tail to be a putative βarr2 recruitment determinant. Lastly, another interesting finding is the conservation of the “SCDS” motif in Lgr4-6 to the V2R and β2AR in a similar locale (Figure 2, *box*), suggesting that this motif, which regulates internalization of Lgr5 may also possess either βarr2 recruitment activity under certain stimuli or act as a priming site required for subsequent phosphorylation of the “TFTSSS” motif. From these data we set out to test the hypothesis that Lgr5 can engage βarr2 through its conserved serine and threonine cluster motifs.

### GRK overexpression drives ligand independent recruitment of βarr2 to Class *B receptors*

**Figure 3:**
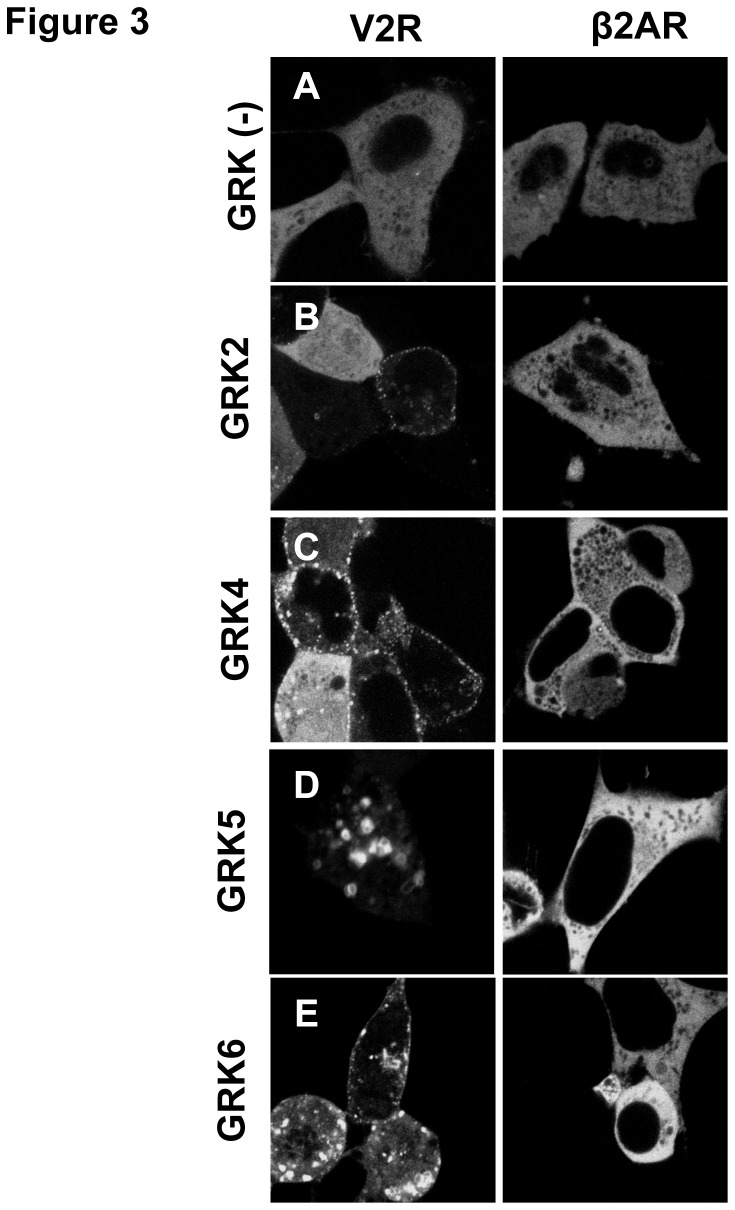
GRK overexpression drives ligand independent βarr2 translocation to Class B but not Class A GPCRs. Typical Class B (V2R, left) or Class A (β2AR, *right*) GPCRs were transiently co-expressed with GFP tagged βarr2 in HEK 293 cells in (A) the absence of GRK overexpression or in the presence of overexpressed (B) GRK2, (C) GRK4, (D) GRK5, or (E) GRK6. Native GFP fluorescence was imaged at 100X with a confocal microscope and representative images presented.

A ligand-independent assay was implemented to test this hypothesis, since Rspondins and Norrin are unable to promote βarr2 translocation [12,17]. The βarr2-GFP translocation assay, and its derivative technologies, have become robust, reliable, and facile assays for assessing GPCR activation [42-44]. On the basis that GRK overexpression can stimulate receptor phosphorylation and receptor sequestration, we sought to determine whether an assay for ligand-independent arrestin recruitment could be implemented using wild-type GRKs [45,46]. As a test for GRK-dependent ligand-independent arrestin recruitment, the prototypical Class B receptor V2R (Figure 3, *Left Panels*) or the Class A β2AR (Figure 3, *Right Panels*) were used. Unstimulated V2R is unable to recruit GFP-tagged βarr2, which remains largely dispersed in the cytoplasm (Figure 3A). In contrast, overexpression of GRK2 or GRK4 in the presence of the V2R can cause translocation of βarr2 to the plasma membrane in the absence of ligand (Figure 3B). Lastly, we found that GRK5 or GRK6 over-expression facilitated recruitment of βarr2 to intracellular vesicles. In contrast, over-expression of GRK2/4/5/6 with the class A β2AR was unable to initiate βarr2 recruitment. These data indicate that this assay can feasibly probe for potential βarr2 interactions, particularly those modulated by serine clusters within the C-tail of a receptor.

### GRK4-6 but not GRK2 overexpression can direct βarr2 recruitment to Lgr5

**Figure 4:**
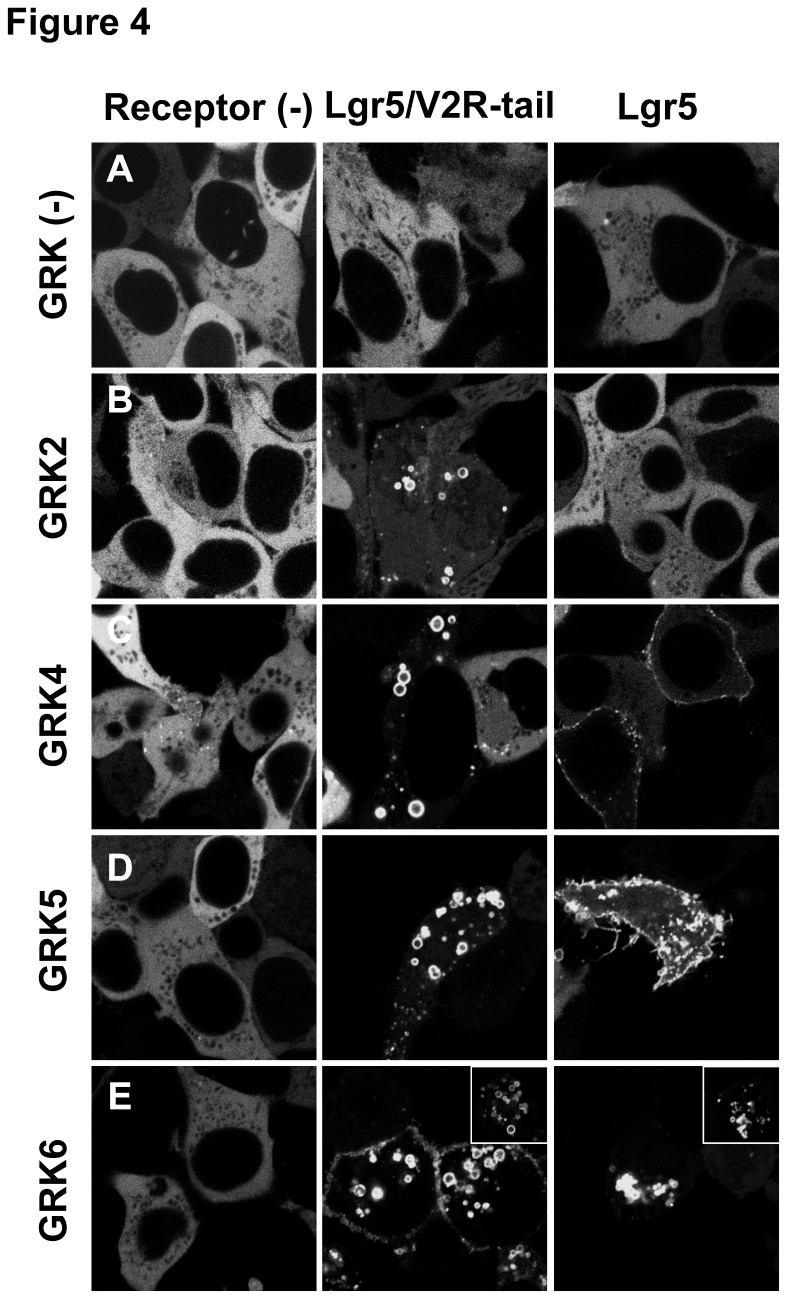
GRK overexpression mediates βarr2 translocation to Lgr5. GFP tagged βarr2 was transiently transfected in HEK 293 cells alone (Left Panels) or in the presence of overexpressed Lgr5/V2R chimera (Middle Panels) or wild-type HA epitope-tagged Lgr5 (Right Panels). βarr2-GFP translocation was visualized using a confocal microscope at 100X in (A) the absence of overexpressed GRK2 or in the presence of overexpressed (B) GRK2, (C) GRK4, (D) GRK5, or (E) GRK6. Inset depicts 2x magnification of images.

To test the effect of GRK on Lgr5, a Lgr5/V2R chimeric receptor generated previously was utilized, (Figure 4A-E *, Middle Panels*) in which the C-tail of Lgr5 was exchanged at amino acid 834 for the V2R C-tail from amino acids 343-371 [24]. In the absence of GRK overexpression, βarr2 remained dispersed in the cytosol (Figure 4A). However, upon overexpression of GRK2/4/5/6 (Figure 4B-E) we found a remarkable and robust recruitment of βarr2 into intracellular vesicles, mirroring activation of a Class B receptor. We repeated these experiments with wild-type Lgr5 to verify that this interaction could be recapitulated with its endogenous tail (Figure 4A-E *, Right Panels*). In both the absence of GRK overexpression or with GRK2 overexpression βarr2 remained localized to the cytosol (Figure 4A,B). In contrast, overexpression of GRK4, GRK5, or GRK6 promoted βarr2 translocation (Figure 4C-E). Interestingly, we observed a continuum of responses that included Class A (membrane) to Class B (vesicular) βarr2 translocation profiles. GRK4 appeared to be the weakest modulator and stimulated a Class A profile with only an occasional cell having appreciable βarr2 vesicular localization. GRK5 possessed moderate activity in which cells with a mix of Class A and Class B βarr2-recruitment could be observed. Finally, GRK6 possessed the strongest activity in this assay and stimulated βarr2 translocation to intracellular vesicles reminiscent of a Class B response. As a control, the overexpression of GRKs only facilitates the translocation of βarr2 when receptors are also overexpresssed (Figure 4A-E *, Left Panels*)*.*

In order to qualitatively assess the dependence of GRK overexpression on βarr2 translocation in the ligand-independent assay, cells with endogenous GRKs or cells transiently transfected and overexpressing GRK5 or GRK6 plasmid were compared. The results confirm that the endogenous levels of GRKs are too low to support ligand-independent translocation of βarr2 to Lgr5 (Figure S1). Ligand mediated activation of GPCRs also promotes βarr1 translocation to the activated receptor, and like βarr2 translocation, it depends upon the molecular determinants in the receptor intracellular domains and C-tail [38]. In the ligand-independent system described, βarr1 translocation to Lgr5 or the V2R does not occur without or with overexpression of GRK2, GRK4, GRK5, or GRK6 (Figure S2). This suggests that Lgr5 either doesn’t recruit βarr1 or as for the V2R, ligand-mediated activation is necessary for this interaction.

**Figure 5:**
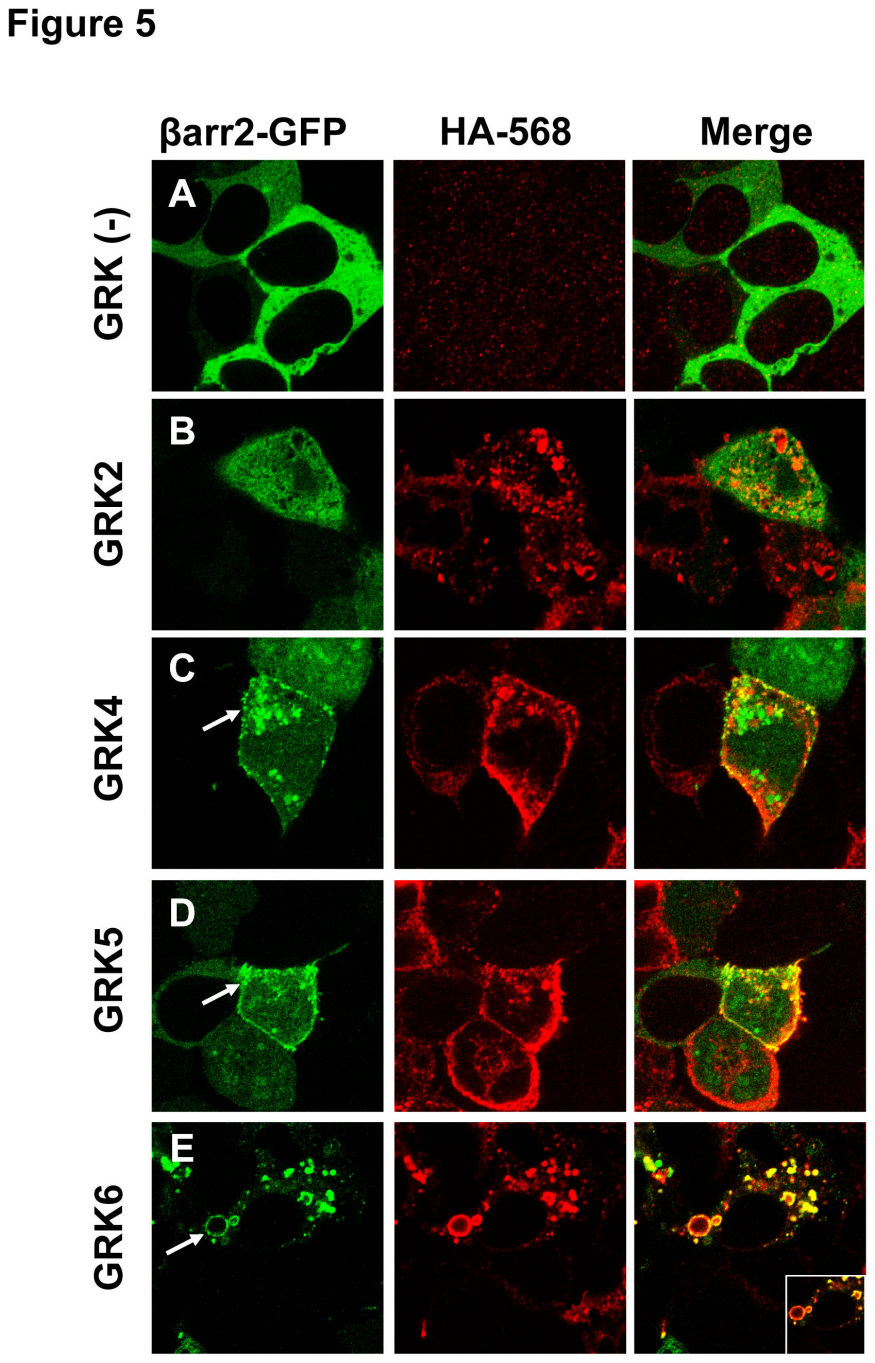
GRK6 overexpression mediates βarr2 recruitment to Lgr5 intracellular vesicles. GFP-tagged βarr2 and HA epitope-tagged Lgr5 was transiently overexpressed in HEK cells (A) without GRK overexpression, or with overexpression of (B) GRK2, (C) GRK4, (D) GRK5, or (E) GRK6. Cells were fixed, permeabilized, and stained with a primary and secondary (568nm) antibody pair to HA. Representative images are presented of native GFP fluorescence (Left Panels, Green), HA-568 (Middle Panels, Red), and merged (Right Panels, yellow denotes colocalization). Arrows point to representative βarr2/Lgr5 colocalization. Inset depicts a 2x magnification.

To verify that βarr2 and Lgr5 are interacting we co-stained for Lgr5 using an antibody directed toward a 3xHA-N-terminal epitope fused to Lgr5 (Figure 5). These data show that Lgr5 is unable to promote translocation of βarr2 to the receptor with overexpressed GRK2, despite the high expression of Lgr5 and its localization to intracellular vesicles (Figure 5A,B),. In contrast, GRK4 and GRK5 are able to facilitate βarr2 translocation to Lgr5 punctae on the plasma membrane (Figure 5C,D). Remarkably, GRK6 overexpression causes βarr2 translocation to large intracellular vesicles that contain Lgr5 (Figure 4E). Collectively, these data are proof-of-principle that structural determinants present in Lgr5 are functionally able to modulate classical GPCR behavioral processes, such as βarr2 recruitment.

### βarr2 recruitment to Lgr5 occurs during internalization and is dependent upon a motif within its C-terminal tail (834-907)

**Figure 6:**
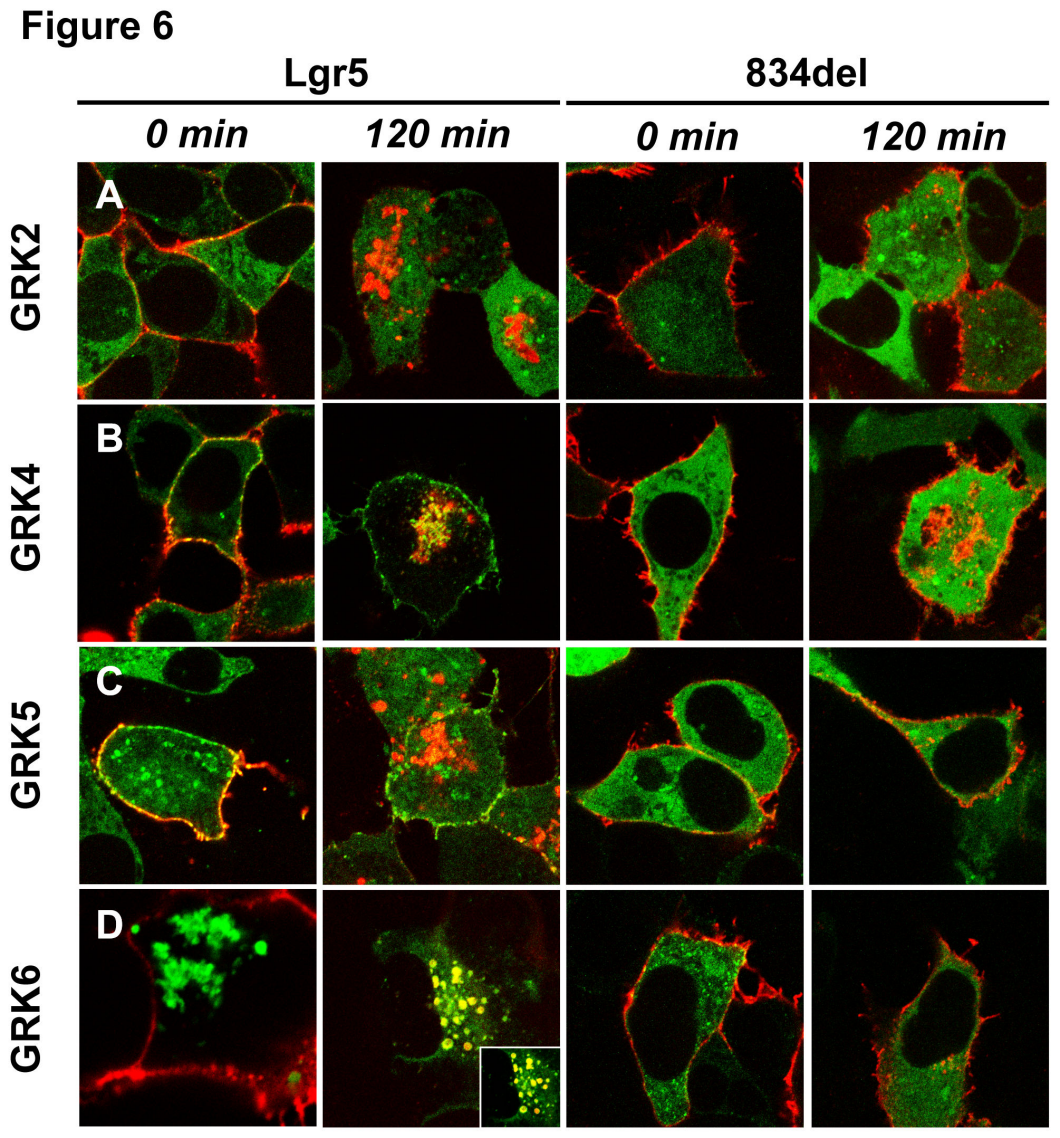
GRK6 overexpression stimulates Class B βarr2 translocation to Lgr5 that is dependent upon a structural determinant in the C-tail. (Left panels) Wild-type HA epitope-tagged Lgr5 or (Right panels) HA epitope-tagged 834del (See Table 1 for truncation site) Lgr5 were cotransfected in HEK293 cells with GFP-tagged βarr2 and (A) GRK2, (B) GRK4, (C) GRK5, or (D) GRK6. Cells were pulsed with an HA antibody on ice, washed, chased, and fixed. Cells were permeabilized and stained with a secondary antibody to the primary HA antibody. Images were collected on a confocal microscope at 100X. (Red:HA and Green:Native GFP were Merged:yellow). Inset depicts 2x magnification.

βarr2 binds to Class B receptors at the plasma membrane and traffics with the receptor during endocytosis into intracellular vesicles [40]. Lgr5 is rapidly internalized to the trans-Golgi network (TGN) following its delivery to the cell surface [24]. Therefore, we sought to determine if the translocation of βarr2 to Lgr5 vesicles occurred with internalized or with de novo synthesized receptors. Lgr5 (N-terminally 3xHA Epitope tagged) and GFP tagged βarr2 were transfected and cells were pulsed with primary antibody, washed, and chased for 0 or 120 minutes to stain surface expressed receptor and follow its trafficking. For wild-type Lgr5 (Figure 6, *A-D, Left Panels*), as expected, we found that GRK2 overexpression didn’t promote βarr2 translocation and colocalization with Lgr5 at the membrane at time 0 minutes nor following Lgr5 trafficking to intracellular vesicles after 120 minutes of chase (Figure 6A). As we have already shown, overexpression of either GRK4 or GRK5 caused translocation of βarr2 to the membrane (Figure 6B,C *, 0 min*). Importantly, this interaction is only transient, as few βarr2/Lgr5 positive vesicles were identified when Lgr5 was allowed to internalize for 120 minutes (Figure 6B,C *, 120 min*). In contrast, in the setting of GRK6 overexpression, a remarkable colocalization of Lgr5 and βarr2 in endocytic vesicles 120 minutes into the chase was found (Figure 6D). 

As further evidence that the principal determinant for this interaction is a component in the C-tail of Lgr5 we also truncated the C-terminus at amino-acid position 834 (834del). In contrast to wild-type Lgr5 the internalization of 834del Lgr5 is largely blunted [24]. Moreover, even in the presence of GRK2/4/5/6, βarr2 fails to translocate (Figure 6A-D respectively). These data indicate that the Lgr5-βarr2 interaction is driven by a protein motif residing in the distal C-tail.

**Table 1:** Overview of constructs utilized in Figures 7 and Figure 8.

**Fig.**	**Receptor**	**Primary Amino Acid Sequence**
	Wild-type	SLRKQTYVWTRSKHPSLMSINSDDVEKQSCDSTQALVTFTSSSITYDLPPSSVPSPAYPVTESCHLSSVAFVPCL
7.1	834del	SL
7.2	864del	SLRKQTYVWTRSKHPSLMSINSDDVEKQSCDS
7.3	875del	SLRKQTYVWTRSKHPSLMSINSDDVEKQSCDSTQALVTFTSSS
7.4	S864A	SLRKQTYVWTRSKHPSLMSINSDDVEKQSCD**A**TQALVTFTSSSITYDLPPSSVPSPAYPVTESCHLSSVAFVPCL
7.5	pDEL 866-907	SLRKQTYVWTRSKHPSLMSINSDDVEKQSCDSTQALV**A**F**AAAA**I**AA**DLPP**AA**VP**A**PA**A**PV**A**E**A**CHL**AA**VAFVPCL
7.6	S873-5A	SLRKQTYVWTRSKHPSLMSINSDDVEKQSCDSTQALVTFT**AAA**ITYDLPPSSVPSPAYPVTESCHLSSVAFVPCL
8.1	pDEL 833-907	**A**LRKQ**AA**VW**A**R**A**KHP**A**LM**A**IN**A**DDVEKQ**A**CD**AA**QALV**A**F**AAAA**I**AA**DLPP**AA**VP**A**PA**A**PV**A**E**A**CHL**AA**VAFVPCL
8.2	pDEL 833-865	**A**LRKQ**AA**VW**A**R**A**KHP**A**LM**A**IN**A**DDVEKQ**A**CD**AA**QALVTFTSSSITYDLPPSSVPSPAYPVTESCHLSSVAFVPCL
8.3	pDEL 844-864	SLRKQTYVWTR**A**KHP**A**LM**A**IN**A**DDVEKQ**A**CD**A**TQALVTFTSSSITYDLPPSSVPSPAYPVTESCHLSSVAFVPCL
8.4	pDEL 844-864 +A844S	SLRKQTYVWTR***S***KHP**A**LM**A**IN**A**DDVEKQ**A**CD**A**TQALVTFTSSSITYDLPPSSVPSPAYPVTESCHLSSVAFVPCL
8.5	pDEL 844-864 +A848S	SLRKQTYVWTR**A**KHP***S***LM**A**IN**A**DDVEKQ**A**CD**A**TQALVTFTSSSITYDLPPSSVPSPAYPVTESCHLSSVAFVPCL
8.6	pDEL 844-864 +A851S	SLRKQTYVWTR**A**KHP**A**LM***S***IN**A**DDVEKQ**A**CD**A**TQALVTFTSSSITYDLPPSSVPSPAYPVTESCHLSSVAFVPCL
8.7	pDEL 844-864 +A854S	SLRKQTYVWTR**A**KHP**A**LM**A**IN***S***DDVEKQ**A**CD**A**TQALVTFTSSSITYDLPPSSVPSPAYPVTESCHLSSVAFVPCL
8.8	pDEL 844-864 +A861/4S	SLRKQTYVWTR**A**KHP**A**LM**A**IN**A**DDVEKQ***S***CD***S***TQALVTFTSSSITYDLPPSSVPSPAYPVTESCHLSSVAFVPCL
8.9	pDEL 844-864 +A861S	SLRKQTYVWTR**A**KHP**A**LM**A**IN**A**DDVEKQ***S***CD**A**TQALVTFTSSSITYDLPPSSVPSPAYPVTESCHLSSVAFVPCL
8.10	pDEL 844-864 +A864S	SLRKQTYVWTR**A**KHP**A**LM**A**IN**A**DDVEKQ**A**CD***S***TQALVTFTSSSITYDLPPSSVPSPAYPVTESCHLSSVAFVPCL
8.11	S861/4A	SLRKQTYVWTRSKHPSLMSINSDDVEKQ**A**CD**A**TQALVTFTSSSITYDLPPSSVPSPAYPVTESCHLSSVAFVPCL
8.12	S861A	SLRKQTYVWTRSKHPSLMSINSDDVEKQ**A**CDSTQALVTFTSSSITYDLPPSSVPSPAYPVTESCHLSSVAFVPCL

Constructs were aligned at amino acid sequence 833. In bold are serine, threonine, or tyrosine residues that were mutated to alanine (**A**). Serines that were returned to wild-type sequence are shown as italicized bold (*S*).

### The “SSS” motif (aa873-875) in Lgr5 is necessary for recruitment of βarr2

**Figure 7:**
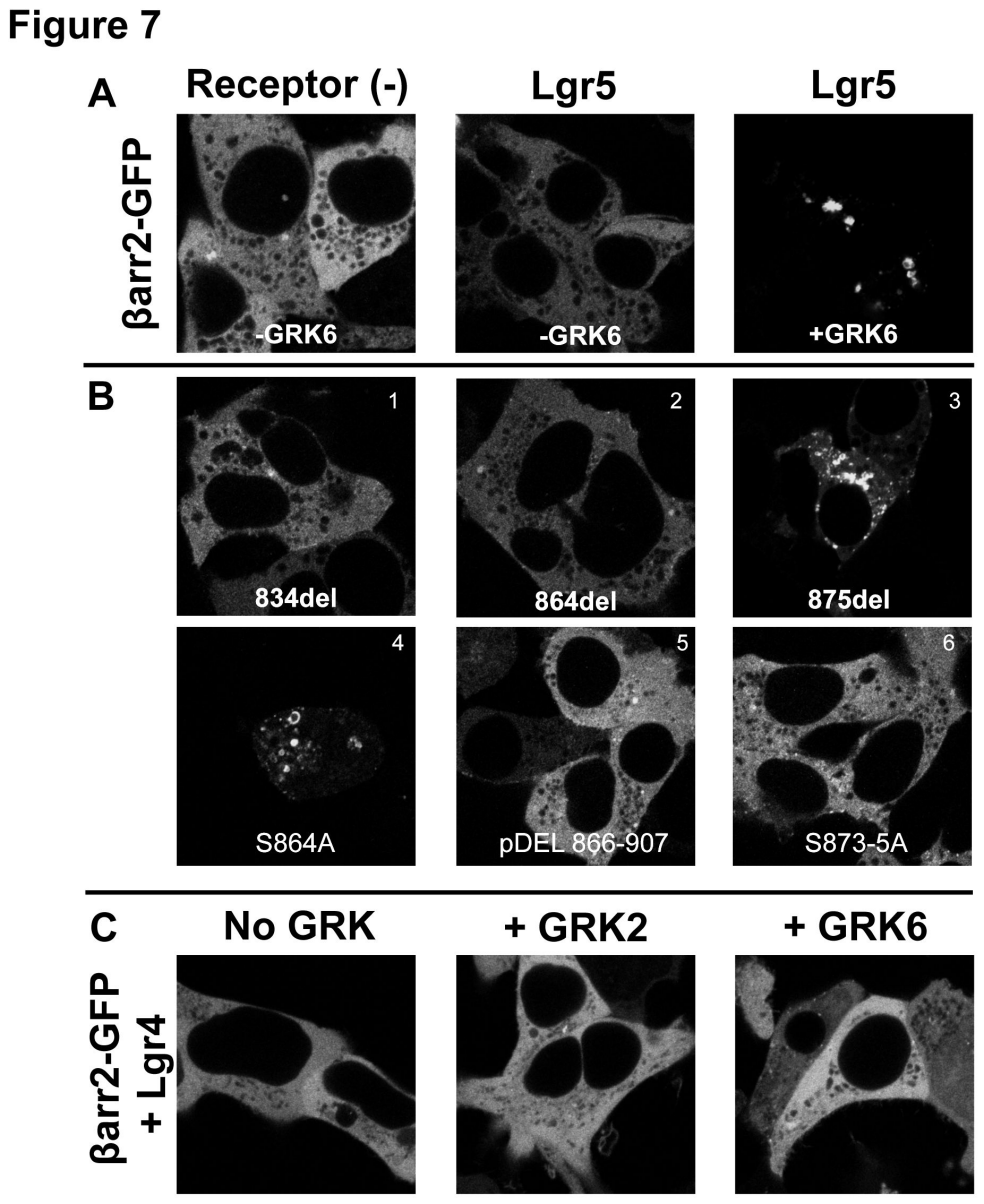
The amino acids “SSS” from position 873-875 in Lgr5 mediate βarr2 translocation. (A) GFP-tagged βarr2 was transiently expressed alone (Left panel) or with wild-type Lgr5/-GRK6 (Middle panel) or Lgr5/+GRK6 (Right panel) in HEK 293 cells. (B) GFP-tagged βarr2 and GRK6 were transiently expressed with the indicated C-tail mutants. The primary acid sequence of the C-tail mutants is provided in tabular form (Table 1 7.1-.6). (C) GFP-tagged βarr2 and Lgr4 were transiently expressed in HEK293 cells in the absence of GRK overexpression or with GRK2 or GRK6 (Left, Middle, and Right panels, respectively). Native GFP fluorescence was imaged by confocal at 100X.

To determine if the threonine and serine cluster in the C-tail acts as a typical GPCR βarr2 translocation motif in Lgr5, βarr2 translocation assays with C-tail truncation mutants were performed. For these studies, we focused on GRK6 driven recruitment to Lgr5 (Figure 7A). Truncations of Lgr5 at amino acid position 864 and 875 were used, based upon the proximity of these residues to Lgr5 internalization motifs and putative βarr2 interacting motifs (Figure 6B). Compared to the truncation at 834, restoring the C-tail to position 864 didn’t facilitate the translocation of βarr2 (Fig. 7*B*.*1, 2*, Table 1). In contrast, truncation of Lgr5 at amino acid position 875 resulted in a receptor still capable of recruiting βarr2 (Fig. 7*B*.*3*, Table 1).

These data provide evidence that an amino acid motif between 865 and 875 is necessary for GRK6 dependent translocation of βarr2 to Lgr5. Near this region is serine 864, previously shown to regulate constitutive internalization of Lgr5. Mutating this serine alone to alanine had no effect (Figure 7*B*.*4*, Table 1), as would be expected based upon Fig. 7*B*.*2*. To determine if phosphorylation could play a role, all potential phosphor-acceptors between amino acid residue 866 and 907 were mutated (pDel 866-907). Results demonstrated that this construct was unable to support βarr2 translocation (Fig.7*B*.*5*, Table 1). The serine/threonine cluster is present within this region (amino acid position 873-875). Therefore, this serine cluster was mutated to alanine. Remarkably, this receptor was also unable to recruit βarr2. In contrast to Lgr5, Lgr4 doesn’t possess this “SSS” motif and as expected doesn’t support GRK mediated βarr2 translocation (Figure 7C). These data demonstrate that that the “SSS” at position 873-875 is required for βarr2 translocation to Lgr5.

### Cooperation between the internalization motif “SCDS” and the “SSS” cluster drives GRK6 dependent βarr2 recruitment to Lgr5

**Figure 8:**
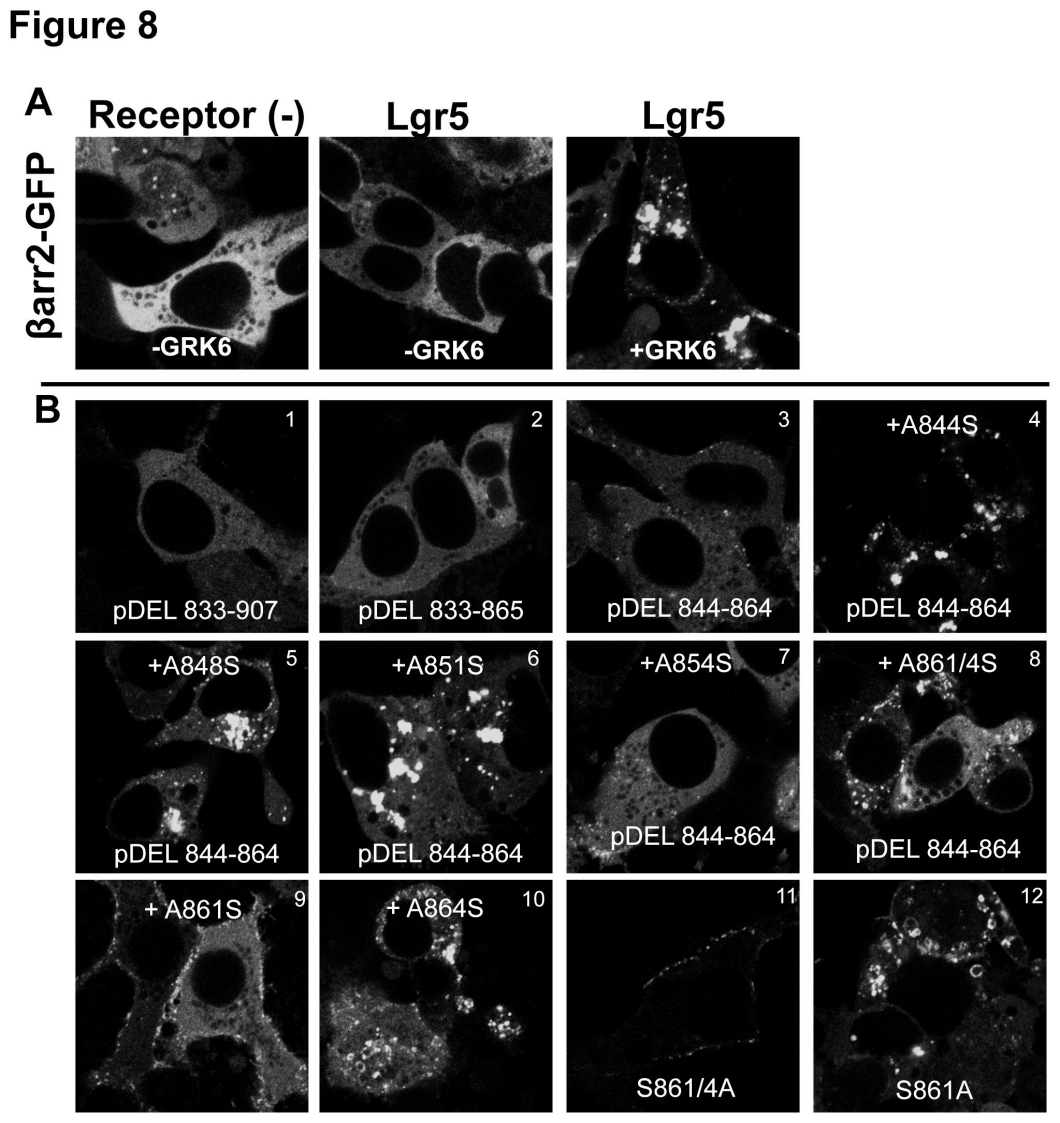
Cooperation of Lgr5 internalization motifs is necessary for “SSS” mediated βarr2 translocation. (A) GFP-tagged βarr2 was expressed in HEK cells without GRK overexpression (Left panel) or co-expresssed with wild-type Lgr5/-GRK or Lgr5/+GRK (Right panel and Middle panel, respectively). (B) GFP-tagged βarr2 was cotransfected with GRK6 and the indicated Lgr5 C-tail mutant. The primary amino acid sequence of mutants are provided in tabular form. (Table 1 8.1-12). Native GFP fluorescence was imaged using confocal microscopy at 100X.

To determine if a motif other than the “SSS” was required for βarr2 recruitment we performed an unbiased screen of all phosphor-acceptors in the C-tail. Alanine substitution mutants were generated at potential phosphorylation sites (Table 1) and were characterized by their ability to recruit βarr2 in a GRK6 dependent manner (Fig. 8*B*.*1-12*) relative to wild-type Lgr5 (Figure 8A). As expected, mutation of all potential phosphorylation sites from amino acid 833 to 907 (pDEL 833-907) inhibited βarr2 recruitment (Fig. 8*B*.*1*). Previously, we have shown that the constitutive internalization of Lgr5 occurs independent of the “SSS” cluster (aa873-875) and βarr2 but rather upon the serine pair 861/864. Therefore, we tested whether these residues or upstream serines were potential priming sites necessary for the functionality of the “SSS” motif. Potential phosphorylation sites from amino acid positions 833-865 (pDEL 833-865) or 844-864 (pDEL 844-864) were mutated to alanine (Table 1). Neither receptor mutant was able to cause translocation of βarr2 (Fig. 8*B*.*2,3*). To identify the phosphorylation sites necessary within 844-864, each site was systematically returned to its wild-type residue using the pDEL 844-864 construct as a template (Table 1). In these experiments, +A844S, +A848S, and +A851S, restored βarr2 translocation activity, while +A854S only weakly restored this activity (Figure 8*B*.*4-7*). These residues were previously shown to be dispensable for internalization, so next we focused on the serine pair at amino acid position 861/864 which robustly affects Lgr5 internalization. Restoration of these residues in tandem (Table 1, Figure 8*B*.*8* or singularly restored βarr2 translocation (Table 1, Figure 8*B*.*9,10*), though restoration at serine 861 didn’t restore the Class B activity. Finally, the necessity of these residues was tested by mutating only these residues in tandem and singularly in an otherwise wild-type receptor. Tandem mutation of 861/4 to alanine markedly reduced βarr2 translocation, eliminating Class B-like vesicular translocation and yet preserving some weak membrane Class A-like activity (Figure 8*B*.*11* and Table 1). In contrast, selective mutation, of either serine 861 or 864, to alanine failed to perturb βarr2 translocation (Figure 8*B*.*12* and Figure 7B.*4* and Table 1). Collectively, these data indicate that the serine motifs present within the internalization motif of Lgr5 (aa844-864) and the “SSS” cluster (aa873-5) act cooperatively to facilitate GRK mediated βarr2 translocation to Lgr5.

### Quantitative analysis of βarr2 translocation to Lgr5 using wide-field high resolution imaging

**Figure 9:**
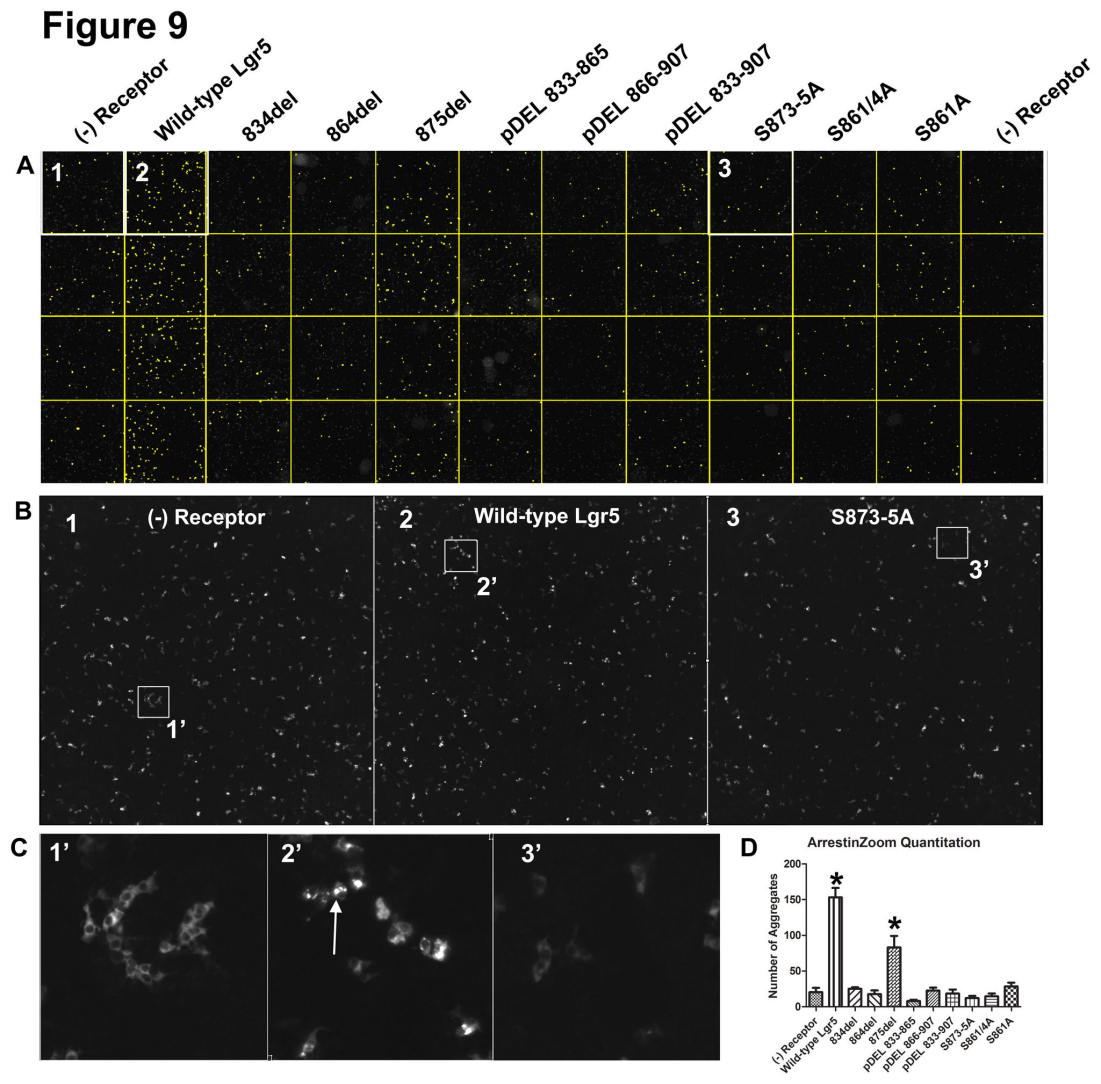
Quantitation of βarr2 recruitment to Lgr5 using an ArrestinZoom Assay (Part 1). GFP-tagged βarr2 and GRK-6 were transiently co-expressed together with the permutations of Lgr5 previously described for confocal analysis and reviewed in Table 1. (A) 48-wells of a 96-well plate were imaged for GFP fluorescence. For each well, 32 images were tiled at 108X magnification and subsequently stitched together. Stitched images were imported into ImageJ and converted to a single montage where each column represents a different experimental condition (as labeled) and each row designates a technical replicate (3.34X Magnification). As described in materials and methods, βarr2-GFP aggregates were identified (highlighted in yellow). (B) Areas outlined in (A) for (1) βarr2-GFP + GRK-6, (2) βarr2-GFP + GRK-6 + Wild-type Lgr5, and (3) βarr2-GFP + GRK-6 + S873-5A at 8.9X magnification (C) Areas outlined in B, presented at 108X magnification (Arrow in 2’ denotes βarr2 aggregates, that are absent without Lgr5 and when the “SSS” cluster is mutated to “AAA”). (D) Highlighted βarr2 aggregates in “A” were quantitated, graphed, and tested for statistically significant differences by 1-way ANOVA and *post* *hoc* Bonferroni correction for multiple comparisons (p<0.05). The results are representative of three independent experiments.

**Figure 10:**
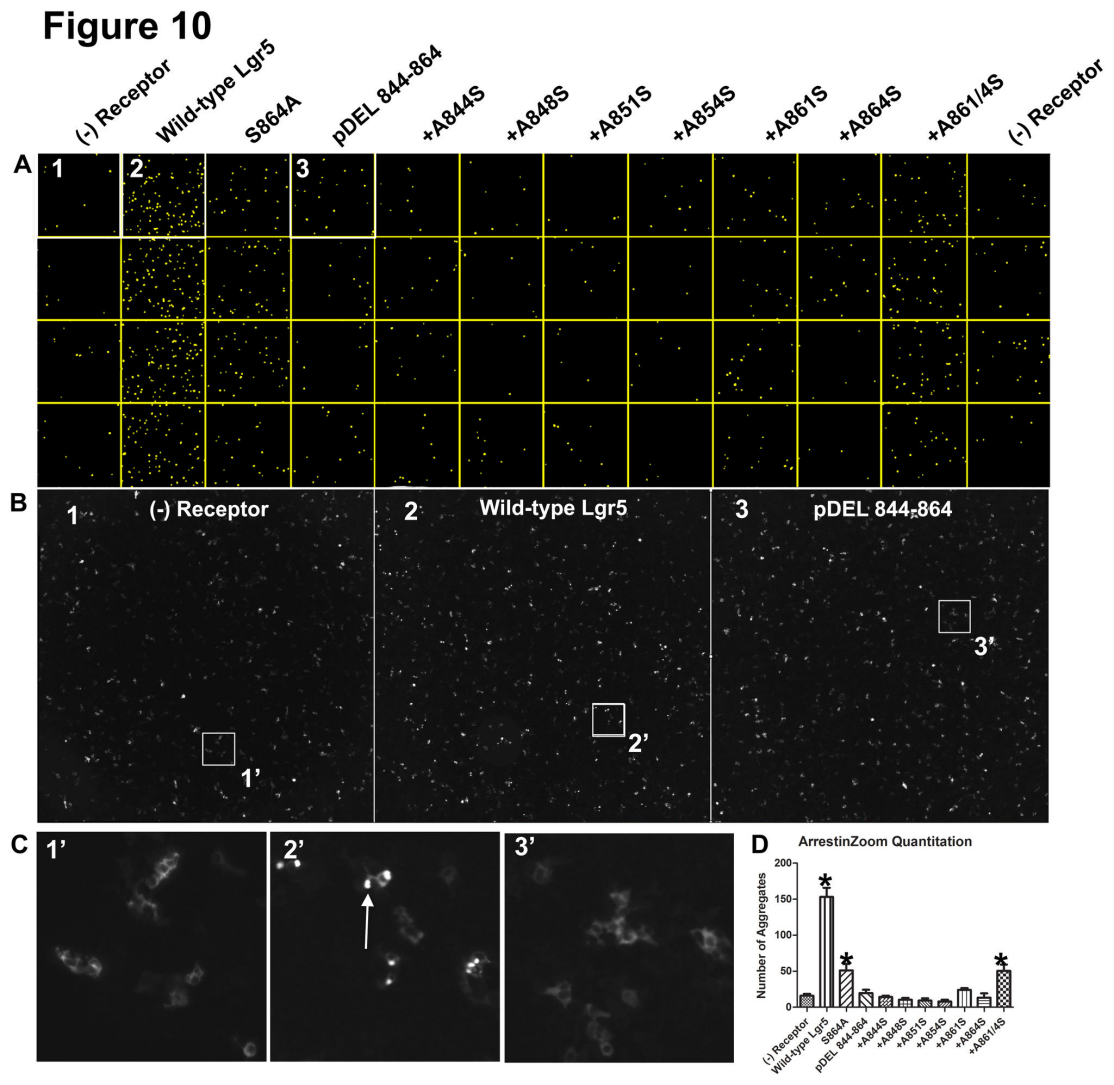
Quantitation of βarr2 recruitment to Lgr5 using an ArrestinZoom Assay (Part 2). GFP-tagged βarr2 and GRK-6 were transiently co-expressed together with the permutations of Lgr5 previously described for confocal analysis and reviewed in Table 1. (A) 48-wells of a 96-well plate were imaged for GFP fluorescence. For each well, 32 images were tiled at 108X magnification and subsequently stitched together. Stitched images were imported into ImageJ and converted to a single montage where each column represents a different experimental condition (as labeled) and each row designates a technical replicate (3.34X Magnification). As described in materials and methods, βarr2-GFP aggregates were identified and are shown in yellow. (B) Areas outlined in (A) for (1) βarr2-GFP + GRK-6, (2) βarr2-GFP + GRK-6 + Wild-type Lgr5, and (3) βarr2-GFP + GRK-6 + pDEL 844-864 at 8.9X magnification (C) Areas outlined in B, presented at 108X magnification (Arrow in 2’ denotes βarr2 aggregates, that are absent without Lgr5 and when the phosphor-acceptors between amino acids 844-864 are mutated to alanine; pDEL844-864). (D) Highlighted βarr2 aggregates in “A” were quantitated, graphed, and tested for statistically significant differences by 1-way ANOVA and *post* *hoc* Bonferroni correction for multiple comparisons (p<0.05). The results are representative of three independent experiments.

To assess βarr2 translocation to Lgr5 in an unbiased manner, a robust and reliable methodology to quantify βarr2-GFP translocation was developed, which we refer to as ArrestinZoom. As opposed to present high-content, medium to high throughput imaging strategies, the ArrestinZoom assay utilizes a relatively inexpensive microscopy platform. This system is capable of providing both low and high resolution wide-field and higher-magnification images using a high numerical aperture zoom lens. When coupled to a low noise CCD camera, motorized stage, and appropriate image acquisition software we were able to acquire thousands of images from a 96-well plate (32images/well = 3072images). The ArrestinZoom assay was applied to all of the Lgr5 constructs previously tested in the confocal translocation assay as discussed in materials in methods. Briefly, multiple tiled images from each well were stitched and then processed in ImageJ to identify βarr2 aggregates. Results of this analysis demonstrated visually that the formation of βarr2 aggregates was dependent upon Lgr5 expression (Figure 9A and 10A) and that the C-tail is critical to this process. Importantly, as the complementary confocal translocation analysis demonstrated, the serine cluster “SSS” from amino acid 873-875 (Figure 9A; Compare Box 3 (S873-5A) to Box 2 (WT)) is necessary for Lgr5-dependent βarr2 translocation and the upstream phosphor-acceptors also play a cooperative role in this process (Figure 10A; Compare Box 3 (pDEL 844-864) to Box 2 (WT)). The boxes outlined in Figure 9A and 10A are also presented at 8.9X (Figure 9B and 10B) and 108X (Figure 9C and 10C) magnification to demonstrate at the cellular level the robust formation of βarr2 aggregates for WT Lgr5 (Box 2, arrow) and their absence in the negative control (Box 1) or phosphor-acceptor mutant (Box 3). Lastly, the identified βarr2 aggregates in 9A and 10A were counted and graphically presented (Figure 9D and 10D). These results demonstrated quantitative and statistical differences in βarr2 aggregate formation for Lgr5 and its mutant derivatives that confirmed the confocal imaging experiments. The results of this study demonstrate for the first time that Lgr5 can recruit βarr2 and begin to identify the molecular determinants of this process.

## Discussion

The signaling pathways potentially engaged by the proposed high-affinity ligands to Lgr4-6 remain enigmatic. Rspondins facilitate Lgr4-6 mediated Wnt-signaling while Norrin only stimulates Lgr4 mediated Wnt-signaling, despite high affinity binding to Lgr5 and 6 [12-17]. Interestingly, each ligand shares a curious inability to engage typical GPCR behavior, such as G protein-coupling or βarr2 recruitment. These observations have caused some to question whether Lgr4-6 are even GPCRs: “Surprisingly, G-protein signaling does not seem to be involved in mediating Lgr5-homolog- derived Wnt signals, raising the question of whether their classification as GPCR's is accurate [10].” 

To fully elucidate the impact that Lgr4-6 signaling plays in cellular behavior, mandates that this question be answered. Therefore, in this study we sought to determine whether Lgr4-6, in particular Lgr5, are able to support classical GPCR behaviors. We demonstrated that Lgr4-6 harbor typical GPCR structural motifs and that Lgr5, in particular, possesses a serine cluster reminiscent of those present in GPCRs capable of high affinity ligand-mediated βarr2 recruitment. Our recent work defined the principal motifs regulating Lgr5 internalization and trafficking to the TGN. Importantly, we demonstrated previously that aa873-875 (“SSS”) are dispensable for internalization [24]. In this study, we show that Lgr5 can recruit βarr2 and that the “SSS” residues are absolutely necessary for this particular process. An unanticipated finding was that βarr2 translocation to Lgr5 also required upstream serines from amino acid 844-864, which together were shown to be essential for the constitutive internalization of Lgr5. These data suggest that these sites act as priming residues for subsequent phosphorylation at 873-875 and that the internalization program must be initiated for recruitment of βarr2. Moreover, they demonstrate that constitutive internalization can be effectively segregated from βarr2-coupling in the absence of a ligand.

We previously concluded that based upon its primary amino acid sequence that either “*the potent β-arrestin binding domain in the Lgr5 tail is a vestigial motif, or more provocatively, it could indicate the existence of another class of endogenous Lgr5 ligands* [24].” Our data provide the first experimental evidence that the “SSS” cluster is more than a vestigial motif and that under the appropriate conditions is a functional βarr2 high-affinity motif supportive of typical GPCR behaviors. 

The GRK overexpression studies presented herein provide interesting insight into the molecular details of this process. Seven mammalian GRKs exist and are classified into 3 groups that include GRK1-like, GRK2-like, and GRK4-like corresponding to GRK1/7, GRK2/3, and GRK4/5/6 respectively [47,48]. The GRK1-like subfamily is expressed in the retina and therefore we only focused on the GRK2- and GRK4-like subfamily. Our studies demonstrated that overexpression of GRK4-like but not GRK2-like family members are able to stimulate recruitment of βarr2 to WT Lgr5. The ability to promote ligand-independent arrestin translocation has been shown previously for a mutant plasma membrane localized and G protein independent form of GRK2 [49]. In contrast, our studies use WT versions of GRKs to demonstrate the agonist-independent translocation of βarr2 to a receptor. The overexpression of GRK4 and GRK6 has also been shown to phosphorylate and cause endocytosis of the β2AR in the absence of ligand [46]. Previous *in silico* findings suggested that the serine cluster “SSS” (aa873-875) in Lgr5 is a likely candidate for GRK phosphorylation [24]. These data, coupled to reports demonstrating that FSHR is tightly regulated by the repertoire of GRKs expressed, further suggest that overexpression of GRK4-like family members phosphorylate Lgr5 and stimulate βarr2 recruitment [5,50].

The disparate activity of GRK2-like and GRK4-like family kinases found in this study further validate previous reports proposing a GRK mediated phosphorylation barcode of GPCRs. Interestingly, while GRK2 and GRK4 family kinases can phosphorylate the same receptor, it has been demonstrated for a few GPCRs that GRK2-like kinases might phosphorylate residues responsible for the desensitization process whereas GRK4-like kinases phosphorylate residues involved in mediating G protein-independent or arrestin mediated signaling [51]. Together with our previous report outlining the molecular determinants of Lgr5 internalization [24], these findings raise the possibility that serines 861/864 might be GRK2-like kinase substrates which mediate endocytosis while the “SSS” (873-875) are the substrates of GRK4-like kinases and are essential for high-affinity βarr2 coupling and downstream signaling. These important concepts will be the subject of future investigations.

The fact that the known high-affinity ligands to Lgr4-6 are unable to stimulate βarr2 recruitment or G protein-coupling is a paradoxical finding given the homology between Lgr4-6 and Lgr1-3 and the ability of Lgr1-3 ligands to elicit typical GPCR behaviors. The GRK overexpression experiments presented in this study suggest Lgr5 is able to support the βarr2 component of typical GPCR behaviors. Interestingly, a handful of other non-GPCRs have shown to be either substrates of GRKs or able to bind arrestins. However, the GPCR class of receptors differ strikingly in their structural characteristics and protein motifs [52,53]. Importantly, Lgr5 possesses the defining structural characteristics and protein motifs inherent to GPCRs and at the appropriate conserved structural locations. In addition to the 7-TM bundle, Lgr5 contains canonical “DRY”, “NPXXY”, and “SSS” serine cluster motifs (Figure 1 and Figure 2). Moreover, during review of our manuscript, a single report was published indicating that Lgr5 may couple to the heterotrimeric G protein alpha subunits G_12/13_ [54]. Therefore, these recent data, together with the multiple lines of structural, biochemical, and cell biological evidence that we present here, indicate that Lgr5 possesses the capacity for classical GPCR behavior. Collectively, these results illustrate that reclassification of Lgr5 as a non-GPCR is premature, and point to a significant knowledge gap in our understanding of Lgr4-6 signaling.

The high-affinity interactions described for R-spondins or Norrin with Lgr4-6 are indisputable, raising the question of what the roles are for each of these ligands in Lgr4-6 biology? The ability to transduce Wnt signaling is intricately regulated by the internalization of Lgr5 and Lgr4 [15,55]. As discussed earlier, we have previously shown that internalization of Lgr5 likely occurs independently of the βarr2 recruitment motif [24]. Therefore, it is altogether likely that Rspondin signaling through Lgr4-6 highlights a unique signaling role that may occur independently of a more typical GPCR signal transduction paradigm. Interestingly, a high-resolution structure of Rspondin-1 with the Lgr4 ectodomain support the notion that Rspondins don’t engage the N-terminus of Lgr4 in a manner conducive to G protein coupling, despite the existence of the necessary structural domains within the receptor [56]. These findings continue to demonstrate the many fascinating aspects of GPCRs including their ability to couple to multiple signaling pathways independent of G proteins or arrestins but shouldn’t discount the existence of alternative ligands or effectors capable of their activation.

The discovery of Norrin as a high-affinity ligand to Lgr4-6 was guided by an evolutionary search for mammalian orthologues of the Drosophila bursicon, a heterodimer of burs/pburs and the ligand for the Drosophila Lgr4 orthologue, *Lgr2*. This analysis revealed that Norrin is closely related to burs/pburs as revealed by its cysteine-knot motif comprised of 11 cysteine residues [17,57]. Like mammalian Lgr1-3 ligands, Drosophila bursicon is only active as a complete heterodimer [57-59]. In contrast Norrin binding and its ability to stimulate Wnt-signaling is independent of heterodimer formation possibly due to its ability to oligomerize [17]. Interestingly, despite binding to Lgr5 and Lgr6, Norrin doesn’t stimulate Wnt-signaling at these receptors. In contrast to Rspondins, Norrin possess a remarkable evolutionary conservation to bursicon [17]. Despite this similarity, the inability to engage G protein or arrestin signaling with Norrin indicates that a component of this pathway is not well understood.

The C-tail alignments demonstrated that Lgr4 and Lgr6 both lack the “SSS” cluster necessary for high-affinity βarr2 recruitment (Figure 2). As expected, we were unable to demonstrate that Lgr4 recruits βarr2 in our ligand-independent GRK assay. The C-tail of Lgr4 is more similar to β2AR than V2R. The β2AR also doesn’t support translocation of βarr2 in our ligand-independent βarr2 assay, presumably since its affinity for βarr2 is too weak. Therefore, using this assay we can’t determine the affinity of βarr2 for Lgr4. For Lgr6, despite much work we are still unable to properly express Lgr6 on the plasma membrane in heterologous cell systems. Instead, we find that Lgr6 continues to be aggregated in the endoplasmic reticulum (ER) (data not shown) indicating that studies investigating Lgr6 signaling need to be carefully evaluated and interpreted. Moreover, Lgr6 completely lacks the “DRY” motif in IC2 suggesting that signaling at Lgr6 may diverge significantly from Lgr4 and Lgr5.

In the course of these studies we developed the ArrestinZoom assay to quickly, reliably, and quantitatively assess βarr2 recruitment to Lgr5 and its mutants. There are currently several techniques in place to quantify βarrestin recruitment in cell assays that for the small laboratory include cost-prohibitive high-content imaging systems, or one dimensional optical readers of bioluminescence energy transfer, enzyme complementation, or transcriptional reporter assays [43,44]. These systems, while effective, can be either expensive or require the generation of assay specific receptor constructs utilizing C-tail receptor/reporter fusions that may alter arrestin affinity. In our analysis, we found the ArrestinZoom assay was able to reliably quantify arrestin recruitment for the majority of constructs tested. The minor discrepancies between it and confocal imaging can probably be resolved using alternative computer algorithms for image processing and utilizing higher resolution cameras. βarr2-GFP translocation to activated GPCRs is a powerful screening tool for the small laboratory. As with other similar methodologies, the potential remains for loss of Arrestin/Receptor affinity due to fusion proteins and additional requirements for validating assays since compounds may conceivably activate βarrestin translocation independent of receptor activation [60]. Even so, the findings with the ArrestinZoom assay, demonstrating that βarr2 recruitment to Lgr5 can be quantified by moderate throughput using high content imaging at relatively low cost, indicate that this approach can significantly enhance the ability of small laboratories to study receptor and protein trafficking.

The potential to directly regulate stem cell activity on the basis of Lgr-targeted therapy is of incredible interest. Unfortunately, the inability to identify endogenous ligands and effectors of Lgr4-6 that support GPCR signaling continues to slow their full biochemical characterization and the search for novel drug therapy. The results of our study demonstrate that Lgr5 possesses the capacity for typical GPCR behaviors. This study is an important first-step in evaluating roles for Lgr4-6 as GPCRs, and provides the rationale for future work, which will explore the missing signaling effectors capable of eliciting these behaviors.

## Supporting Information

Figure S1 **GRK overexpression is required for βarr2 translocation to Lgr5.** (A) GRK5 and (B) GRK6 antibody staining. As described in materials and methdods, HEK293T cells were plated and transfected with 3xHA-Lgr5 or 3xHA-834del Lgr5, βarr2-GFP, and GRK5 or GRK6, as indicated at left. Cells were fixed, permeabilized, stained, and imaged for the HA epitope (Blue, 633nm secondary dectection), GRK5 or GRK6 (Red, 568nm secondary detection), βarr2-GFP (Green, Native fluoresence). A merge of all three channels is presented at the right. Endogenous GRK5 or GRK6 are below the levels of antibody detection and are unable to promote ligand-independent βarr2-GFP translocation. Overexpression of GRK5 or GRK6 enabled translocation of βarr2 to Lgr5 that was depedent upon the Lgr5 C-tail.
(TIF)

Figure S2 **Lgr5 does not recruit βarr1 in the ligand-independent translocation assay.** HEK293T cells wereplated on 35mm glass-bottom dishes (MatTek: P35G-0-10C) and transfected with wild-type human V2R or Lgr5 (2μg), βarr1-GFP (1.5μg), and [+/- (2μg)] GRK2, GRK4, GRK5, or GRK6 as indicated. Cells were imaged live at 100X by confocal microscopy (βarr1-GFP, Green). (A) In a control experiment, V2R and βarr1-GFP transfected cells were imaged live before and 25 minutes after 10μM vasopressin stimulus (AVP: Sigma V9879). As expected, AVP stimulus of V2R caused translocation of βarr1-GFP to vesicles.(B) In contrast to the ligand-independent GRK assay described in the manuscript, GRK overexpression alone is unable to promote βarr1-GFP translocation for the V2R or Lgr5.
(TIF)

File S1 **Class 1 GPCR Alignment.**
(TXT)
