# Correction: The Stem Cell-Expressed Receptor Lgr5 Possesses Canonical and Functionally Active Molecular Determinants Critical to β-arrestin-2 Recruitment

**DOI:** 10.1371/annotation/7f83735d-53d4-42f3-b3b2-b05dd5db059c

**Published:** 2014-01-10

**Authors:** Joshua C. Snyder, Lauren K. Rochelle, Larry S. Barak, Marc G. Caron

The authors inadvertently omitted a grant from the Funding statement. The correct Funding is:

This work was supported in part by a Duke Cancer Center Stewart Trust and Duke Cancer Center Cancer and the Environment Grant to JCS, LKR, LSB, and MGC; NIDA P30 5P30DA29925 to LSB and MGC, NIH IMAT Program 1R21CA173245 to LSB and MGC, and a NIMH F32 NRSA 5F32MH092013-03 to JCS. The funders had no role in study design, data collection and analysis, decision to publish, or preparation of the manuscript.

