# Supplementary figures and images for: The Stem Cell-Expressed Receptor Lgr5 Possesses Canonical and Functionally Active Molecular Determinants Critical to β-arrestin-2 Recruitment
Source: PLoS One. 2013 Dec 27;8(12):e84476. doi: 10.1371/journal.pone.0084476 (PMC3873998; doi:10.1371/journal.pone.0084476)

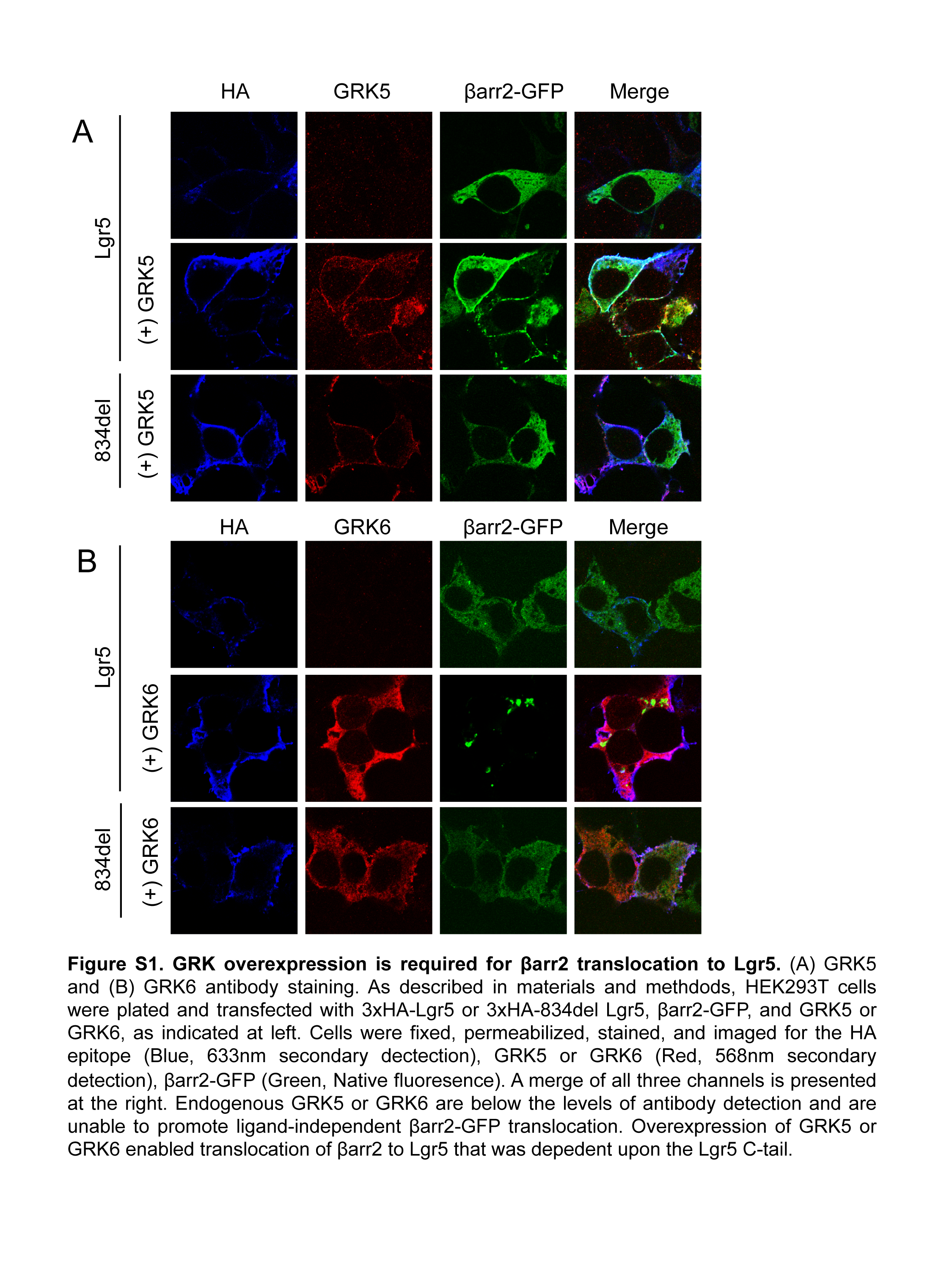

Supplement: Figure S1 — GRK overexpression is required for βarr2 translocation to Lgr5. (A) GRK5 and (B) GRK6 antibody staining. As described in materials and methdods, HEK293T cells were plated and transfected with 3xHA-Lgr5 or 3xHA-834del Lgr5, βarr2-GFP, and GRK5 or GRK6, as indicated at left. Cells were fixed, permeabilized, stained, and imaged for the HA epitope (Blue, 633nm secondary dectection), GRK5 or GRK6 (Red, 568nm secondary detection), βarr2-GFP (Green, Native fluoresence). A merge of all three channels is presented at the right. Endogenous GRK5 or GRK6 are below the levels of antibody detection and are unable to promote ligand-independent βarr2-GFP translocation. Overexpression of GRK5 or GRK6 enabled translocation of βarr2 to Lgr5 that was depedent upon the Lgr5 C-tail. (TIF) [file pone.0084476.s001.tif]

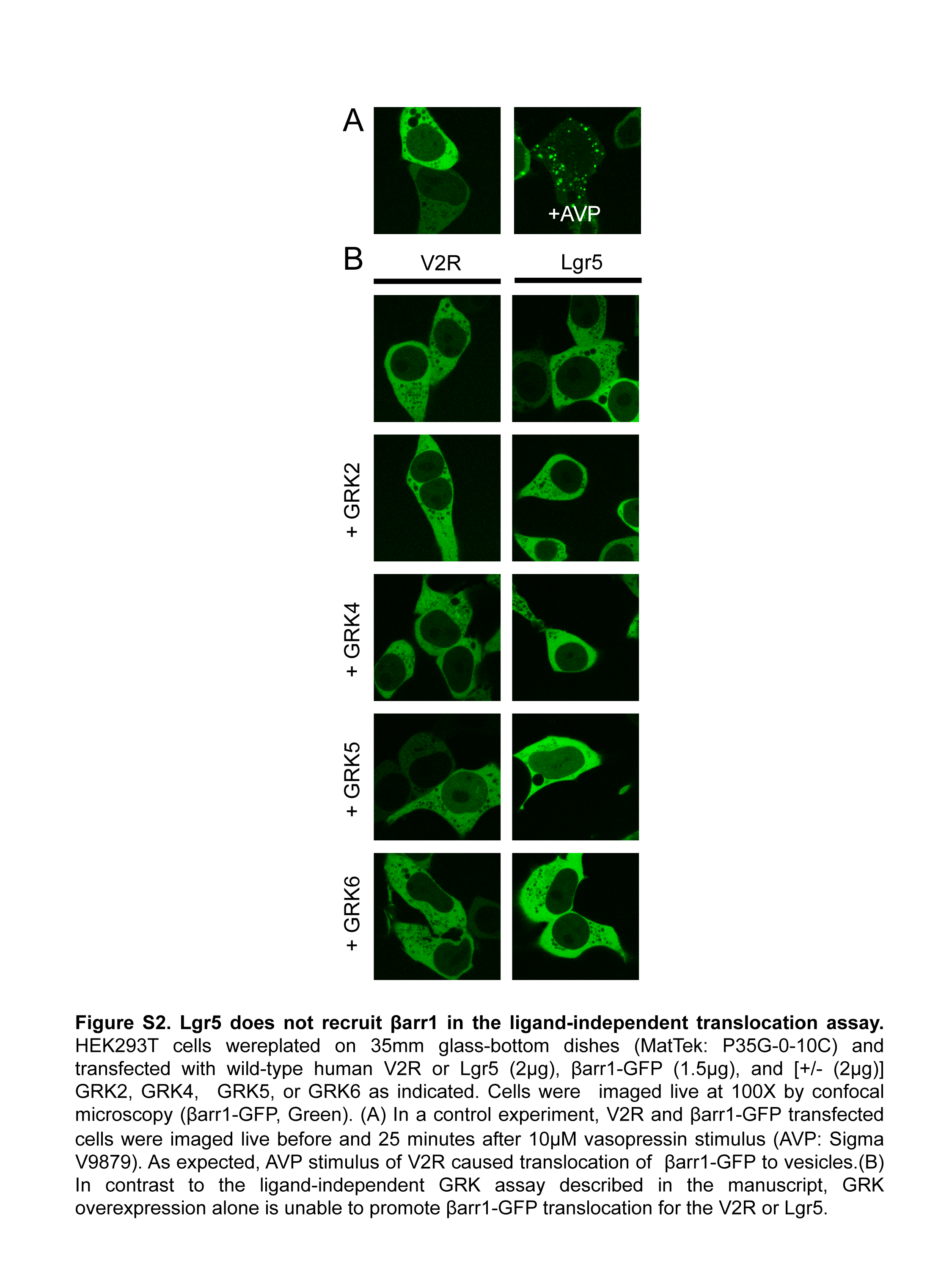

Supplement: Figure S2 — Lgr5 does not recruit βarr1 in the ligand-independent translocation assay. HEK293T cells wereplated on 35mm glass-bottom dishes (MatTek: P35G-0-10C) and transfected with wild-type human V2R or Lgr5 (2μg), βarr1-GFP (1.5μg), and [+/- (2μg)] GRK2, GRK4, GRK5, or GRK6 as indicated. Cells were imaged live at 100X by confocal microscopy (βarr1-GFP, Green). (A) In a control experiment, V2R and βarr1-GFP transfected cells were imaged live before and 25 minutes after 10μM vasopressin stimulus (AVP: Sigma V9879). As expected, AVP stimulus of V2R caused translocation of βarr1-GFP to vesicles.(B) In contrast to the ligand-independent GRK assay described in the manuscript, GRK overexpression alone is unable to promote βarr1-GFP translocation for the V2R or Lgr5. (TIF) [file pone.0084476.s002.tif]
